# Commentary: Pilot study of the adaptation of an alcohol, tobacco, and illicit drug use intervention for vulnerable urban young adults

**DOI:** 10.3389/fpubh.2023.1203699

**Published:** 2023-07-21

**Authors:** Steve Sussman, Artur Galimov, Leah Meza

**Affiliations:** ^1^Department of Population and Public Health Sciences, Keck School of Medicine, Institute for Health Promotion and Disease Prevention, University of Southern California, Los Angeles, CA, United States; ^2^Department of Psychology, University of Southern California, Los Angeles, CA, United States; ^3^School of Social Work, University of Southern California, Los Angeles, CA, United States

**Keywords:** adaptation, Project TND, pilot study, immediate outcomes, urban young adults

## Introduction

Project Towards No Drug Abuse (TND) is an effective, evidence-based, drug abuse prevention program that has provided favorable effects in 7 out of 7 cluster-randomized controlled trials (1). Ferguson and colleagues (2) attempted to provide a pilot test of an adapted version of Project TND. This study was conducted among 30 initially enrolled African American youth aged 16 to 21 years of age, who participated from January of 2016 through July of 2017. The study involved collaboration of the Louisiana State University Health Science Center-New Orleans Comprehensive Alcohol-HIV/AIDS Research Center (CARC) with the Youth Empowerment Project (YEP) in New Orleans. While partnering with a community-based social service organization to assess the feasibility of this program with ethnic minority youth and young adults is to be applauded, the authors should have been more careful not to speculate beyond what the data suggests, as they did.

## Methods, results, and discussion

This was an immediate outcomes evaluation of adapted program content with a novel population (location and age group) and revised implementation schedule of Project TND. While the authors mentioned that no behavioral effect was observed (as indicated in the last sentence of their Abstract, for example), one should not expect a behavioral impact at an immediate posttest. All one can successfully measure in an immediate outcome evaluation of a prevention program for youth is program implementation fidelity, including dosage, adherence, quality of program delivery, participant responsiveness or acceptance, and knowledge gains from the program (3). In fact, overall receptivity of the program (i.e., student responsiveness to the program) was high (the average rating across intervention sessions was 9.5 out of 10, SD = 0.3). Nonetheless, to evaluate the efficacy of the intervention, there must be evidence of significant effects for at least one long-term follow-up at an appropriate interval beyond the end of the intervention (4–7). The authors needed at minimum a 6-month (preferably 1-year) follow-up to evaluate the program efficacy (4–7). This was also a single-group design study and (relative) efficacy trials require a rigorous research design with at least one control condition that does not receive the tested intervention (4).

## Population and implementation

The small sample size (*n* = 30) and extremely low retention rate were also major limitations. The sample size needs to be large enough to allow for sufficient statistical power to conduct meaningful efficacy analyses (4). Of the 30 participants enrolled in this study, only 8 (26.7%) completed both the pre- and immediate post-intervention surveys. Also, the intervention had 3 cohorts of participants recruited over a 1$\frac{1}{2}$ year period (3 months each cohort; 12 weeks of programming). This would indicate that individual group size was smaller than the developer's recommended group size, especially considering absenteeism in implementation (which was not disclosed by authors). Further, the age range of participants included in this study was 16–21 years (mean = 18.3, SD = 1.3), while the original TND program was normed on an age group of 14–19 years (i.e., mean = 16.8, SD = 0.8), a mean of 2 years younger (1). There is simply not enough power to conduct any formal analyses or to measure much of anything other than program receptivity. Despite these limitations, all participants that completed the immediate post-test survey (*n* = 8; 100%) in Ferguson et al. study (2) reported they were not intending to use other drugs within the next 12 months after the intervention (vs. 87.5% at pre-intervention), which implies potential efficacy of Project TND.

Implementation fidelity, or the degree to which a program is delivered as intended by the developers, is one of the major factors in the success of research-based prevention programs like Project TND (8). One of the main reasons why implementation fidelity is important is that it may help to explain why interventions succeed or fail (8). The original Project TND considered four key domains of implementation fidelity (9): (a) dosage (the amount of the program received by students, assessed via attendance records), (b) adherence (extent to which the guidelines of the program were followed), (c) quality of delivery (how well the educator implemented the program), and (d) responsiveness of participants. While overall student responsiveness to the program (i.e., receptivity) was high in this study, attendance (only 23.3% of participants completed >75% of the intervention sessions) and retention rates were very low (26.7%).

### Program content changes

Authors also describe re-ordering, and abbreviating/combining program content. Sessions 1 and 2 (Listening and Stereotyping), and Sessions 7 and 9 (Marijuana Panel and Tobacco Basketball) were condensed into 1-hour session periods, and the study evaluation measures (pretest and posttest) were added as part of the program, which turned the 12-session, 4-week TND program into an adapted 14 session, 3-month program. This is not the original implementation design of the Project TND program (1).

While external Project TND trainers delivered an on-site group training prior to the start of the project, all modifications/adaptations to the Project TND curriculum were not formally reviewed and/or approved. The purpose of Project TND implementation training is to provide implementation staff with the skills needed to deliver program content with fidelity, and to provide implementers with an understanding of the content, instructional techniques, and objectives of the program. Program developers and/or certified Project TND trainers do not formally approve adaptations to program material, especially without sufficient data to the support the change.

## Conclusions

Since the Project TND program was delivered as a 12-week program (one session per week) to an older population with programming content adaptations, this can destroy the evidence-base. In fact, the Project TND website provides guidance on program implementation, stating minimum delivery is 2 sessions per week (over a 6-week period) for favorable results, and specifically that one session per week is not recommended (10).

There was no explicit use of a systematic adaptation approach in the process described in the Ferguson study. The authors could have referred to systematic adaptation frameworks like the ADAPT-ITT framework (11), which includes eight phases (assessment, decision, administration, production, topical experts, integration, training, and testing) highlighting the integration of certified trainers as experts in the adaptation process. Another adaptation model is the chain model, which includes six steps: theory, pooling, perceived efficacy studies, immediate impact studies, pilot testing, and immediate impact/mediator measures development (12). This model includes a great deal of input from the potential young participants.

In conclusion, it is quite clear that fidelity of programming being demonstrated in this study is lacking for the variety of reasons presented. While the authors of the article were complimentary to the TND program in some ways, they made speculations far above what the data collected could provide, particularly considering the vast limitations. Making any claims about preventive alcohol, tobacco, or other drug use (ATOD) behavior based on their methodological design is simply not possible. Future work should be careful to be more cautious of any potentially causal statements made.

## Author contributions

All authors took an active role in the manuscript. SS drafted the manuscript. AG and LM provided critical revision to the manuscript. All authors approved the final submitted manuscript.
